# Enzymatic Hydrolysis of Poly(3-Hydroxybutyrate-*co*-3-Hydroxyvalerate) Scaffolds

**DOI:** 10.3390/ma13132992

**Published:** 2020-07-05

**Authors:** Adriana Kovalcik, Stanislav Obruca, Michal Kalina, Michal Machovsky, Vojtech Enev, Michaela Jakesova, Marketa Sobkova, Ivana Marova

**Affiliations:** 1 Department of Food Chemistry and Biotechnology, Faculty of Chemistry, Brno University of Technology, Purkynova 118, 612 00 Brno, Czech Republic; obruca@fch.vut.cz (S.O.); xcjakesova@vutbr.cz (M.J.); xcsobkovama@vutbr.cz (M.S.); marova@fch.vut.cz (I.M.); 2 Department of Physical and Applied Chemistry, Faculty of Chemistry, Brno University of Technology, Purkynova 118, 612 00 Brno, Czech Republic; kalina-m@fch.vut.cz (M.K.); enev@fch.vut.cz (V.E.); 3 Centre of Polymer Systems, Tomas Bata University in Zlin, trida Tomase Bati 5678, 760 01 Zlin, Czech Republic; machovsky@utb.cz

**Keywords:** degradation kinetics, grape pomace, hydrolysis, mechanical properties, morphology, scaffolds, polyhydroxyalkanoates, thermal properties

## Abstract

Polyhydroxyalkanoates (PHAs) are hydrolyzable bio-polyesters. The possibility of utilizing lignocellulosic waste by-products and grape pomace as carbon sources for PHA biosynthesis was investigated. PHAs were biosynthesized by employing *Cupriavidus necator* grown on fructose (PHBV-1) or grape sugar extract (PHBV-2). Fifty grams of lyophilized grape sugar extract contained 19.2 g of glucose, 19.1 g of fructose, 2.7 g of pectin, 0.52 g of polyphenols, 0.51 g of flavonoids and 7.97 g of non-identified rest compounds. The grape sugar extract supported the higher production of biomass and modified the composition of PHBV-2. The biosynthesized PHAs served as matrices for the preparation of the scaffolds. The PHBV-2 scaffolds had about 44.2% lower crystallinity compared to the PHBV-1 scaffolds. The degree of crystallinity markedly influenced the mechanical behavior and enzymatic hydrolysis of the PHA scaffolds in the synthetic gastric juice and phosphate buffer saline solution with the lipase for 81 days. The higher proportion of amorphous moieties in PHBV-2 accelerated enzymatic hydrolysis. After 81-days of lasting enzymatic hydrolysis, the morphological changes of the PHBV-1 scaffolds were negligible compared to the visible destruction of the PHBV-2 scaffolds. These results indicated that the presence of pectin and phenolic moieties in PHBV may markedly change the semi-crystalline character of PHBV, as well as its mechanical properties and the course of abiotic or enzymatic hydrolysis.

## 1. Introduction

In recent years, changes in the climate, quick drawing of the global resources, and plastic and microplastic pollution have evoked significant efforts for changes in society. The ongoing changes are supported by the systematic implementation of EU directives and rules. A ban on single-use plastics by 2021 can be considered as a start of the transformation of a consumable society into a sustainable circular and resource-efficient economy. Therefore, the demand for the larger volume production of biobased and biodegradable polymers is growing. However, the producers of biopolymers are facing now several challenges, such as (1) the high costs of input sources and production that results in expensive biodegradable polymers, (2) the utilization of sugars as substrates commonly used in the food industry, and (3) a low oil price that further limits the affordability of biodegradable polymers on the market.

One of the options on how to increase the attractiveness of biodegradable polymers might be to incorporate the circular economy into production and valorize lignocellulosic waste. This work focuses on the biosynthesis of polyhydroxyalkanoates (PHAs) utilizing the lignocellulosic waste substrate derived from wine production. PHAs are polyesters of 3-hydroxyalkanoic acids and belong to biobased, biodegradable, and biocompatible polymers. These polymers are synthesized and accumulated by numerous prokaryotes in the form of intracellular granules. The biological function of these materials in bacterial cells is the storage of carbon and energy, as well as the enhancement of stress robustness of the bacterial cells [1]. Generally, PHAs can be produced by approaches of microbial biotechnology and extracted from bacterial cells. After that, they can find applications in water treatment (denitrification) [2], paper modification (sizing of paper) [3], cosmetic industries, pharmaceutical industries (biosurfactants and drug delivery systems) [4,5], prosthetics and medical devices production [6,7,8], medical applications (tissue engineering) [9], and packaging [10]. PHAs are thermoplastics with a large scale of properties depending on the monomeric composition of polymers or copolymers. The structure of a PHA can be modified by varying bacterial strains and feeding precursor substrates [11]. Compared to conventional non-biodegradable thermoplastics, the biggest advantages of PHA utilization are their renewable origin, biodegradability, and biocompatibility. The products of PHA decomposition are 3-hydroxylkanoic acids that are common metabolites in the human body. The low-molecular-weight homopolymers of 3-hydroxybutyrate are components of many biological cells [12]. Additionally, PHAs are known as non-teratogenic [13] and non-carcinogenic [14] materials. PHAs are considered to be emerging non-hazardous materials for various medical applications such as stents, drug delivery systems, impacts, sutures, nerve repair and regeneration, and artificial heart valves [8,15].

Polymeric scaffolds with engineered properties are widely used in tissue engineering; their role is to act as templates for tissue regeneration in order to guide the growth of new tissue [16]. PHAs are considered to be auspicious materials for scaffold fabrication. For instance, Puppi et al. utilized a copolymer of 3-hydroxybutyrate and 3-hydroxyhexanoate for the preparation of scaffolds using mixtures with different chloroform/ethanol ratios, which provided scaffolds characterized by tunable dual-scale porosity, and the resulting microporosity affected cell proliferation [17]. Wei et al. recently developed and described highly porous PHA microspheres that could be used as injectable scaffolds for tissue regeneration [18]. PHA scaffolds can also be formed by micro- and nanofibers and generated by various spinning techniques [19,20] and 3D printing technologies [21,22].

The stability of scaffolds and the safety of decomposition products are critical issues that should be considered in tissue engineering. PHAs are generally regarded as relatively stable materials in human-body conditions. PHAs are effectively degraded by specific enzymes called PHA depolymerases; however, these enzymes are typical for microorganisms capable of PHA synthesis and/or degradation [23]. There is no PHA depolymerase activity in human body fluids or tissues. Hence, PHAs cannot be used as materials for applications requiring the rapid biodegradation of materials such as self-absorbing sutures. On the other hand, PHAs are very prospective materials for medical applications requiring the stability and slow decomposition of materials. However, PHAs can be non-specifically degraded by some ubiquitous hydrolases such as lipases, which are omnipresent enzymes in the human body environment [24]. The course of PHA degradation depends on several parameters. These include the polymer’s chemical composition, molecular weight, ratio of amorphous and crystalline phases, sample morphology, and degradation conditions. It should be noted that variations in the molecular weight values of polymers significantly affect the course of their degradation and influence the changes in their physical properties (morphology, mechanical properties, and semi-crystalline character) [25]. Wu et al. reported that amorphous PHA copolymers containing 4-hydroxybutyrate are much more prone to hydrolysis than 3-hydroxybutyrate homopolymers or copolymer 3-hydroxyvalerate [9].

The most investigated PHAs are poly-3-hydroxybutyrate (PHB) and poly(3-hydroxybutyrate-co-3-hydroxyvalerate) (PHBV). The work’s objective was (1) to investigate properties of PHBV biosynthesized by *Cupriavidus necator* using fructose and grape sugar extract as carbon sources and (2) to compare the thermal properties, mechanical properties, and abiotic/enzymatic hydrolysis of scaffolds based on commercial PHB and produced PHBV. The use of waste materials like grape pomace for the biotechnological synthesis of PHAs is important, especially from the secondary use of waste resources from agriculture, thus decreasing the polymer’s cost. However, waste carbon sources are not pure substances and contain many admixtures. The impurities present in waste carbon materials may influence the accumulation of PHA in bacteria as well as the properties of the accumulated polymers. Therefore, in addition to the determination of biomass and polymers yields, it is also essential to monitor the composition and properties of the produced polymers for their desired application.

## 2. Materials and Methods

### 2.1. Microorganism and Materials

*Cupriavidus necator* H16 (CCM 3726–Czech Collection of Microorganisms, Brno, Czech Republic) was purchased from the Czech Collection of Microorganisms (Brno, Czech Republic).

The grape sugar extract was recovered from the ground seedless Müller Thurgau grape pomace (collection after harvest October 2017, dehydration, and pressing from the Reva Rakvice winery, Rakvice, Czech Republic) by enzymatic extraction with the addition of 5 wt% Viscozyme L (Sigma Aldrich, St. Louis, MO, USA) and lyophilization. The prepared lyophilized grape sugar extract was kept at 4 °C before its use. Fifty grams of lyophilized grape sugar extract contained 19.2 g of glucose, 19.1 g of fructose, 2.7 g of pectin, 0.52 g of polyphenols 0.51 g of flavonoids and 7.97 g of non-identified rest compounds.

Poly(hydroxybutyrate) (PHB Hydal) was provided by Nafigate Corporation, Prague, Czech Republic.

### 2.2. Production of PHBV in a Bioreactor

Fermentations with *C. necator* were performed in a 2-L bioreactor (BioFlo/CelliGen 115, New Brunswick, Eppendorf AG, Hamburg, Germany) using 20 g L^−1^ of fructose or 50 g L^−1^ of lyophilized grape sugar extract as a growth substrate and valeric acid as a precursor. The initial volume of the production media was 1.2 L for the bioprocess and 130 mL of a 24-h *C. necator* inoculum grown in the same cultivation medium. The production media contained 3 g of (NH_4_)_2_SO_4_, 1 g of KH_2_PO_4_, 11.1 g of Na_2_HPO_4_.12 H_2_O, 0.2 g of MgSO_4_, 1 mL of a microelement solution, a carbon source (growth substrate), and 1 L of distilled water. The composition of the microelement solution was described by Obruca et al. [26]. The media, microelement solution, and carbon source were autoclaved before use at 121 °C for 20 min. The fermentation conditions were as follows: 30 °C, pH 7, 30% D.O. (dissolved oxygen) level (which was automatically controlled by the regulation of stirring), aeration at 1.0 L min^−1^, and a cultivation time of 36 h. The pH was controlled at pH 7 with a 1 M H_2_SO_4_ solution and a 30% NaOH solution. The valeric acid was fed separately at the moment when the concentration of the produced biomass reached about 3 and 5 g L^−1^. The additional nitrogen and carbon sources were fed independently if necessary (ammonium sulfate was maintained at 3 g L^−1^ and fructose was kept at 20 g L^−1^ (or 50 g L^−1^ in the case of lyophilized grape sugar)). The small 10 mL volumes of the medium were withdrawn during cultivation for the determination of biomass and PHB concentration. The biomass after cultivation was harvested by centrifugation at 8000 rpm for 10 min.

### 2.3. Preparation of Scaffolds

First, PHAs were extracted from the lyophilized cells with hot chloroform and precipitated in ethanol (10-fold access). The precipitated sample was lyophilized. The scaffolds were prepared by the solution casting of the lyophilized PHAs in hot chloroform. Chloroform was evaporated for two days in the hood, and the formed scaffolds were peeled off and further dried under a vacuum for 24 h.

### 2.4. Degradation of Scaffolds in Model Fluids

The degradation rate of the scaffolds (a 10 mm scaffold diameter with a thickness of 0.15 mm) was studied by their incubation at 37 °C for 81 days in (A) synthetic gastric juice (SGJ) prepared according to Atkins and Peacock (pH 1.6 and pepsin 0.7 U mg^−1^) [27] and a (B) phosphate buffer saline solution (PBS) with lipase (0.1 g L^−1^, pH 7.4). (Lipase Aspergillus oryzae, 8.6 U mg^−1^, Mucos Pharma, Unterhaching, Germany) [28]. The model fluids were changed twice during the degradation experiment. The progress in degradation was monitored by the periodic measurement of residual mass weight, average molecular weight (M_n_ and M_w_), and the scaffolds’ polydispersity (Đ) in time.

### 2.5. Analytical Procedures

The cell dry weight (CDW) was determined gravimetrically after centrifugation, purification with distilled water, and the drying of 10 mL of the sample. The PHA content was analyzed by gas chromatography after the methanolysis of the dried cells (Trace G.C. Ultra, Thermo Scientific, Waltham, MA, USA) as described previously [29]. The residual concentrations of reduced nitrogen NH_4_^+^ were determined spectrophotometrically (Spectrophotometer Helios Delta, Thermo Spectronic) at 450 nm by a reaction with the Nessler reagent [30]. Fructose and glucose contents were estimated by liquid chromatography (pump LCP 4020, thermostat LCO 101, degasser DG1210, and refractometer detector RIDK 102; Ecom, Czech Republic) with a REZEX-ROA column (150 × 4.6 mm, 5 µm; Phenomex, Torrance, CA, USA).

The molecular weights and polydispersities of the scaffolds before and during the degradation were determined by a size exclusion chromatographer (SEC, Infinity 1260, Agilent Technologies, Santa Clara, CA, USA) coupled to a multiangle laser light scattering (MALLS) detector (Dawn Heleos II, Wyatt Technology, Santa Barbara, CA, USA) and a differential refractometer (dRI, Optilab T-rEX, Wyatt Technology, Santa Barbara, CA, USA). The PHB and PHBV samples were solubilized in HPLC-grade chloroform (4 mg mL^−1^) for 3 h at 70 °C and filtered before the analysis through a syringe filter (nylon membrane with a pore size 0.45 μm). The SEC separation was performed by a PL (Polar) gel MIXED-C column (300 × 7.5 mm, Agilent Technology, Santa Clara, CA, USA) in a mobile phase (HPLC-grade chloroform) flow rate and pre-filtered through a 0.02 μm membrane filter at 0.6 mL min^−1^. The molecular weight and polydispersity of PHB and PHBV were determined with the ASTRA software (Wyatt Technology, version 6.1) by using the values of refractive index increment (0.0336 mL g^−1^), as determined from the differential refractometer response assuming a 100% recovery of freshly prepared samples of PHB and PHBV from the column.

Before and after degradation, the FTIR spectra of scaffolds were obtained employing an attenuated total reflectance (ATR) technique using a Nicolet iS50 spectrometer (Thermo Fisher Scientific, Waltham, MA, USA). This measurement was taken at room temperature (in an air-conditioned room) on the built-in diamond ATR crystal. The FTIR spectrum was recorded over the range of 4000–400 cm^−1^ at a 4 cm^−1^ resolution and represented an average of 128 scans. An ATR crystal in the ambient atmosphere (air) was used as the background for infrared measurement. The raw absorption spectrum was evaluated with no artificial processing (e.g., baseline corrections, ATR corrections, or atmospheric suppression).

The mechanical properties of the solution-casted scaffolds were measured by an Instron 3365 (Instron, Norwood, MA, USA). The specimens were dumbbell-shaped with a parallel length of 50 mm, a grip section width of 4 mm, and a thickness of 0.15 mm. The gauge length was set to 20 mm. The applied strain rate was 1 mm min^−1^. Average values of Young’s modulus (E), tensile stress at maximum (σ_B_), and tensile strain at break (ε_B_) were calculated from stress/strain plots of five specimens.

Differential scanning calorimetry (DSC) was applied to determine the thermal properties of the scaffolds before and after degradation in model fluids. Samples of approximately 4 mg were placed into sealed aluminum pans and analyzed under a constant nitrogen flow of 50 mL min^−1^ in a DSC instrument (DSC Q 2000, T.A. Instruments, New Castle, DE, USA). The heating cycle was run from 25 to 190 °C at a heating rate of 10 °C min^−1^. Furthermore, samples were kept for two minutes at 190 °C and cooled to −50 °C from the melt at a cooling rate of 10 °C min^−1^.

The morphology of the scaffolds before and after degradation was studied by thermionic emission scanning electron microscopy (ESEM) (VEGA II LMU, TESCAN). The specimens were prepared by the cryogenic fracturing of the investigated scaffolds in liquid nitrogen and then coated with a thin layer of Au/Pd. The microscope equipped with the SE (secondary electron) detector was operated in a high-vacuum mode at an acceleration voltage of 5 kV.

## 3. Results

### 3.1. Production of PHBV and Properties of PHB, PHBV-1, and PHBV-2 Scaffolds

**Figure 1:**
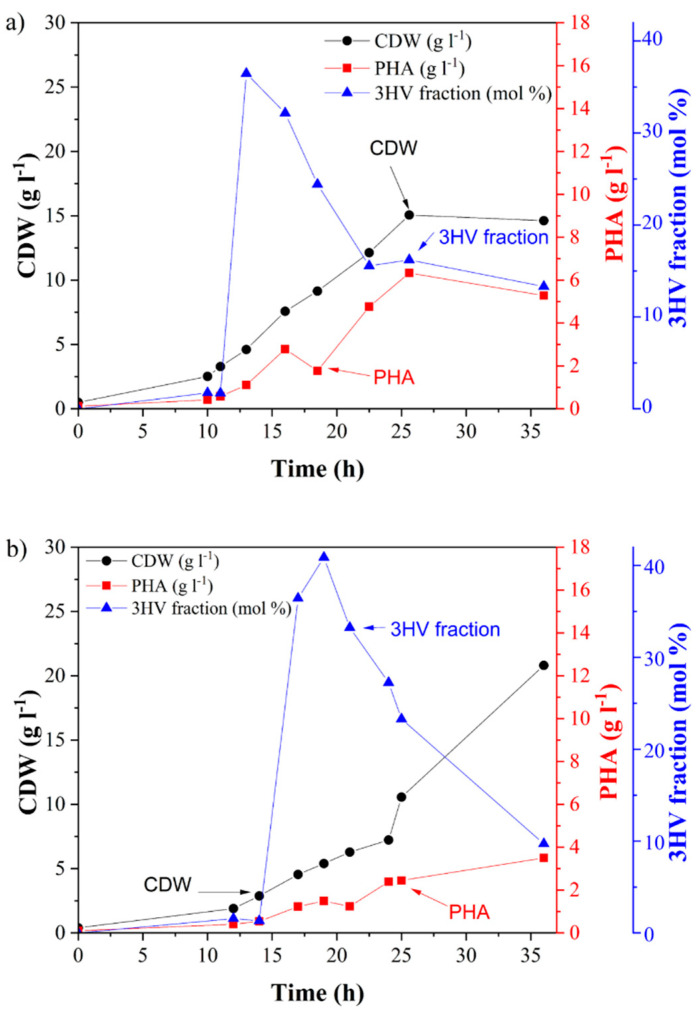
Growth and production characteristics of *Cupriavidus* *necator* grown on (**a**) fructose and (**b**) grape sugar extract.

PHB was obtained as a commercial product, and PHBV was produced by biosynthesis in a laboratory 2-L bioreactor. Two kinds of PHBV were prepared: (1) PHBV-1 was produced by the cultivation of *C. necator* on fructose and valeric acid, and (2) PHBV-2 was obtained by using grape sugar extract and valeric acid as carbon sources. Grape pomace is a cheap and abundant by-product of wine production. It is a lignocellulosic material with a high content of residual sugars, in particular glucose and fructose. In this work, we prepared grape sugar extract from grape pomace by enzymatic hydrolysis and used it as a carbon source for the cultivation of *C. necator*. PHBV synthesis was supported by the addition of valeric acid as a precursor. The courses of both fermentations are shown in Figure 1. In both cases, PHBV was accumulated in the biomass. The obtained biomass and PHBV-1 titers were 14.6 and 5.3 g L^−1^, respectively, when pure fructose was used as a substrate. The content of the 3-hydroxyvalerate fraction (3HV) in PHBV-1 was 13.3 mol %. Interestingly, when the using grape sugar extract, the concentration of biomass yields markedly increased. Still, the accumulated PHA content was substantially lower than in the case of using fructose as the substrate (Table 1). The final PHA concentration in PHBV-2 was 3.5 g L^−1^ with a 9.7 mol % 3HV fraction. This indicates that grape pomace contained substances that supported the growth of the bacterium’s growth without the necessity to accumulate PHAs. It is likely that some nitrogen-rich compounds (such as proteins or peptides) present in the grape pomace extract supported biomass growth but suppressed PHA biosynthesis since it is well known that PHA synthesis is stimulated by nitrogen limitation in *C. necator* [31]. It should be pointed out that the final molar fraction of 3HV was comparable in both copolymers: 13.3% in PHBV-1 and 9.7% in PHBV-2.

**Figure 2:**
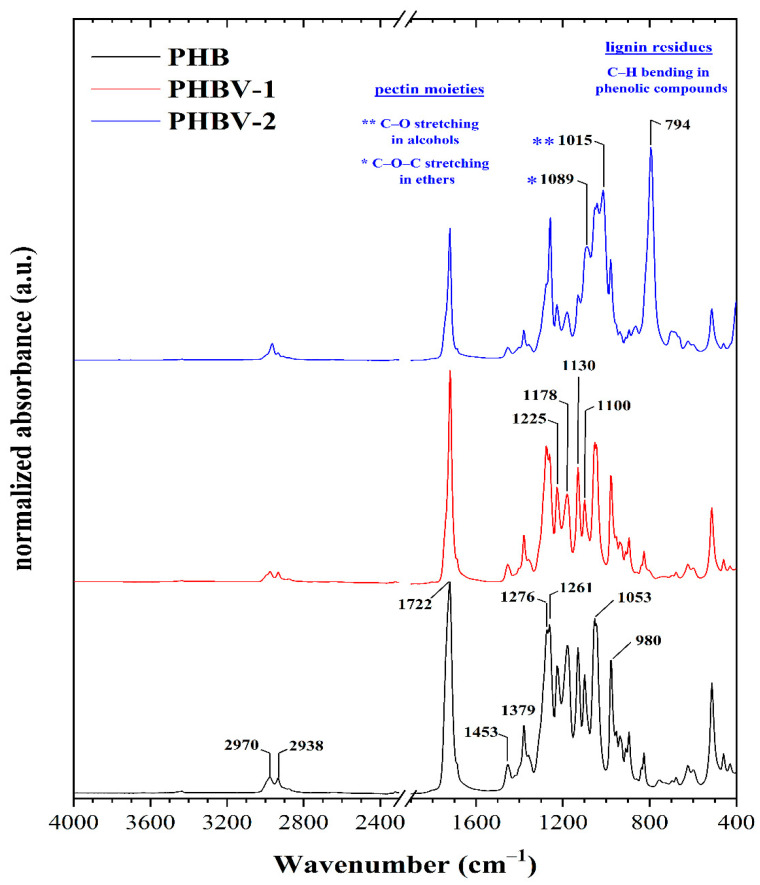
Attenuated total reflectance (ATR)-FTIR spectra of PHB, PHB-1, and PHB-2.

Both materials, PHBV-1 and PHBV-2, were extracted from biomass and, along with commercial PHB, further characterized, and used for scaffold preparation. The ATR-FTIR technique was used for a more profound structural characterization of the synthesized PHB and PHBV copolymers. The ATR-FTIR spectra of the PHB and PHBV copolymers are presented in Figure 2, and the interpretation of their absorption bands was conducted according to literature data [32,33,34,35,36,37]. The attribution of the significant infrared peaks of the PHB and its copolymers (i.e., PHBV-1 and PHBV-2) that were observed in the ATR spectra are presented in Table 2.

The spectra of all samples showed several standard spectral features. One of these was represented by a sharp absorption band with variable intensity at around ~1722 cm^−1^, attributed to symmetric C=O stretching in aliphatic esters. The presence of aliphatic ester groups were usually also manifested by sharp and intensive bands centered at about ~1261 cm^−1^ and ~1053 cm^−1^ that resulted from C–O and C–C–O stretching vibrations mode. Moreover, the presence of these functional groups was revealed by a sharp band at 1178 cm^−1^ ascribed to the C–O–C stretching of saturated alkyl-esters. A band at about 1225 cm^−1^, preferentially attributed to the C–O stretching of the aliphatic ester was more apparent in the PHB sample. Furthermore, the absorption band located at 1100 cm^−1^ was attributed to the stretching vibration of the O–C–C moieties [33]. The presence of the esters’ functional groups was confirmed by the bands at 980 cm^−1^ that could be assigned to the stretching C–O–C groups.

The absorption bands at about 1722, 1276, 1225, and 980 cm^−1^ were assigned to the stretching vibration of the crystalline phase of PHB and its copolymers. On the other hand, the amorphous phase in the samples was indicated by the bands centered at about 1178 and 1130 cm^−1^ resulting from the asymmetric and symmetric stretching vibrations of the C–O–C groups [33]. The degree of the crystalline phase that is known as the crystallinity index was determined using the *I*_C–O_/*I*_–CH2–_ ratio (the ratio of the intensity at 1225 cm^−1^ to the intensity at 1453 cm^−1^). The value of this ratio (Table 3) was lowest for the PHBV-2 copolymer. The intensity ratio showed that the relative content of the amorphous phase’s relevant content was highest for PHBV-2 (i.e., most moderate content of crystalline phase), whereas that of the PHB sample was the lowest. In other words, the PHB sample was characterized by a higher crystallinity of the scaffold. Furthermore, this calculated index suggested that PHBV-1 was characterized by a slightly higher content of the crystalline phase compared to the second copolymer.

Both PHB and PHBV-1 showed the presence of aliphatic hydrocarbons in the range of 3000–2800 cm^−1^ and 1480 –1300 cm^−1^. The intense band at 2970 cm^−1^ and the weak band at 2938 cm^−1^ indicate asymmetric and symmetric C–H stretching of the methyl groups. Two C–H deformation bands were found in the second region. Similarly, the bands at 1453 cm^−1^ and 1379 cm^−1^ belong to the asymmetric and symmetric stretching of the –CH_2_– and –CH_3_ groups. The relative content of aliphatic hydrocarbons was similar in all the samples.

The spectra confirmed that PHBV copolymers were produced. However, the composition of PHBV-2 differed slightly from PHBV-1. These differences were mainly visible in the fingerprint region in the range of 1100–700 cm^−1^. Aside from the already discussed ester groups, the presence of additional oxygen-containing functional groups could also be derived from the ATR spectra. In addition to the characteristic PHBV absorption bands, it seems that pectin and phenolics residues absorption bands appeared. The bands at 1089 and 1015 cm^−1^ indicated pectin moieties. These were attributed to the C–O–C stretching in ethers and to the C–O stretching of primary alcohols. Furthermore, the spectrum of PHBV-2 contained a sharp and intensive absorption band at 794 cm^−1^ corresponding to the C–H bending in phenolic compounds [38]. Therefore, it can be concluded that the composition of PHBV-2 was modified by these substances from grape pomace co-isolated from the biomass.

**Figure 3:**
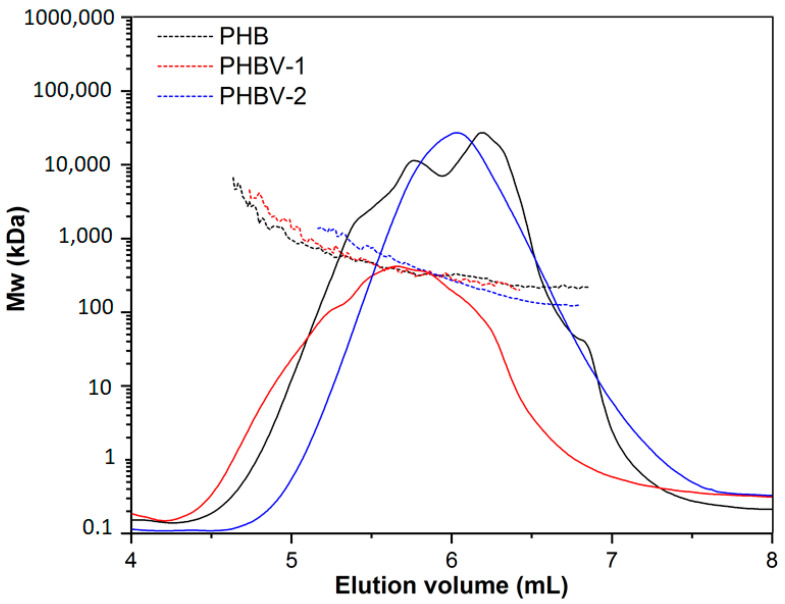
Size exclusion chromatography-multiangle laser light scattering (SEC-MALLS) chromatograms of PHB, PHBV-1, and PHBV-2.

SEC-MALLS was used as a general technique for the routine characterization of molecular weight, molecular size, and polydispersity of the used PHA. SEC-MALLS chromatograms are shown in Figure 3. The molecular weight values of the tested polymers were in the following order: PHBV-1 (Mw = 517 ± 11.08, **Đ** = 1.1) > PHB (Mw = 480 ± 4.22, **Đ** = 1.5) > PHBV-2 (Mw = 235 ± 5.33, **Đ** = 1.3). Small differences in the molecular weights of PHB and PHBV-1 did not indicate any particular differences in the degradation of these two polymers. However, the molecular weight of PHV-2 was much lower than that of PHB and PHBV-1. Therefore, the degradation of PHBV-2 in model fluids could be expected to proceed differently.

**Figure 4:**
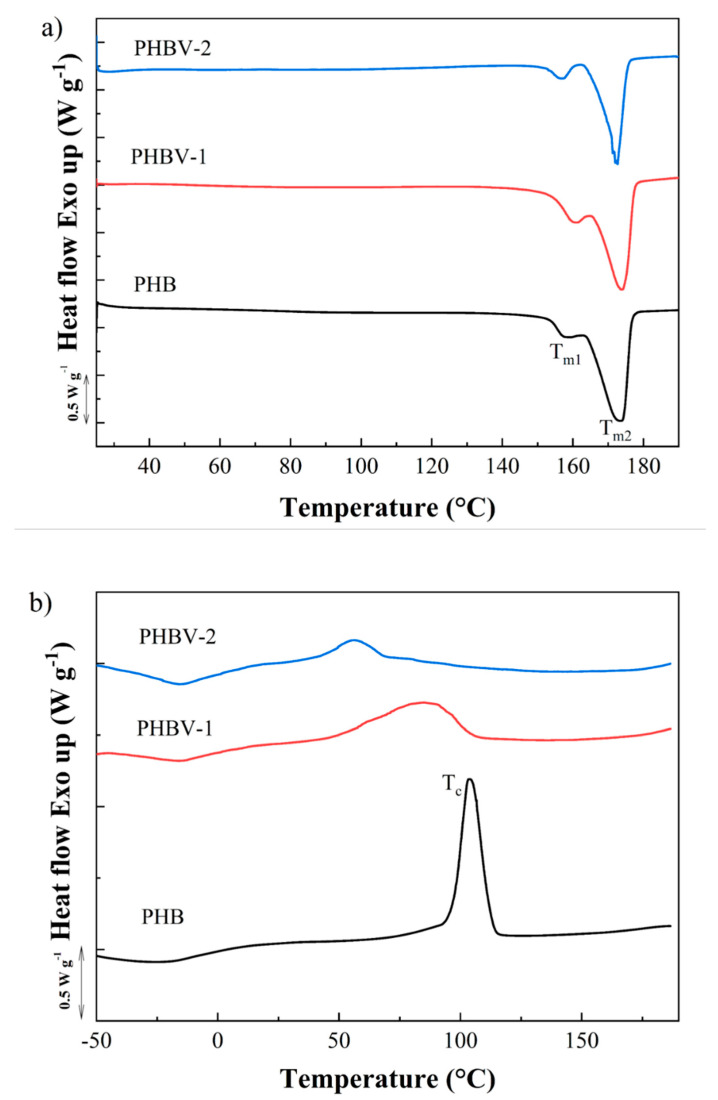
Differential scanning calorimetry (DSC) (**a**) first heating and (**b**) subsequent cooling scans for the PHB, PHBV-1, and PHBV-2 scaffolds.

The melting and crystallization behavior of PHB, PHBV-1, andPHBV-2, were studied by DSC (Figure 4). First, heating scans obtained at the rate of 10 °C min^−1^ showed double melting behavior for all tested PHAs (Figure 4a). Two melting peaks at approximately 163 and 173 °C indicated the presence of crystals with different temperature stabilities, the form of which may be explained by the double lamellar thickness population model [39]. The values of melting enthalpy were detected as follows: PHBV-1 (ΔH_m_ = 81.7 J g^−1^) > PHB (ΔH_m_ = 76.3 J g^−1^) > PHBV-2 (ΔH_m_ = 45.6 J g^−1^). The crystallinity degree presented by PHBV-2 was about 40.2–44.2% lower compared to those detected for PHB and PHBV-2. These results indicated that impurities co-isolated with the PHBV-2 copolymer decreased the crystallinity of the materials. The crystallization kinetics of PHBV-1 and PHBV-2 after melting for both copolymers differed in comparison to the PHB polymer. The crystallization peak for PHB was clearly detected at 103.7 °C with ΔH_c_ = 74.7 J g^−1^. PHBV-1 displayed the crystallization peak at a much lower temperature compared to PHB. Moreover, the large peak indicated slower crystallization kinetics (T_c_ = 85.0 °C and ΔH_c_ = 57.4 J g^−1^). The crystallization of PHBV-2 was even more delayed, showing the crystallization peak at 56.4 °C with ΔH_c_ = 46.5 J g^−1^.

**Figure 5:**
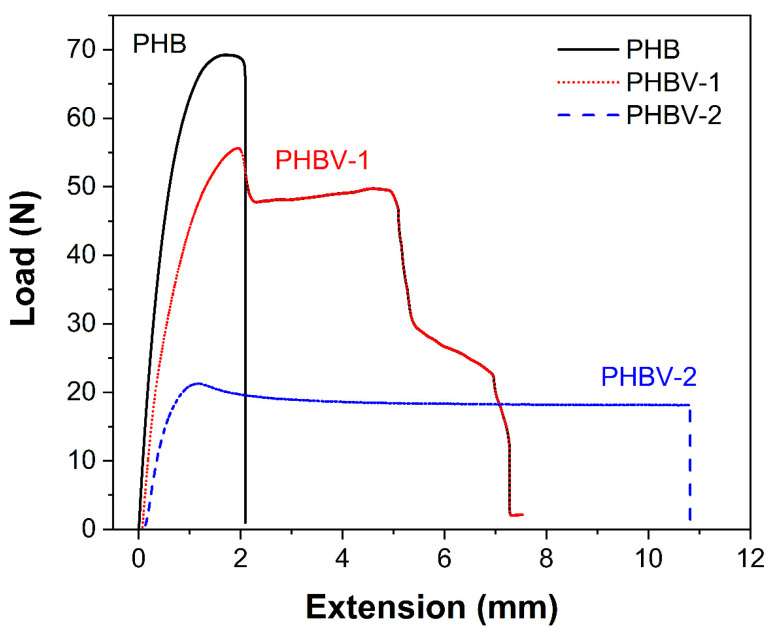
Load versus extension curves for the PHB, PHBV-1, and PHBV-2 scaffolds.

Figure 5 and Table 3 show the mechanical properties of the PHB, PHBV-1, and PHBV-2 scaffolds. The high crystalline PHB films displayed a much higher tensile strength and E-modulus than the PHBV films. These results were in relation to the semi-crystalline nature of PHBV samples. PHBV-2 films were more ductile and elastic compared to PHBV-1 films, thus correlating with lower crystallinity.

### 3.2. Degradation of PHB and PHBV Scaffolds in Model Fluids

**Figure 6:**
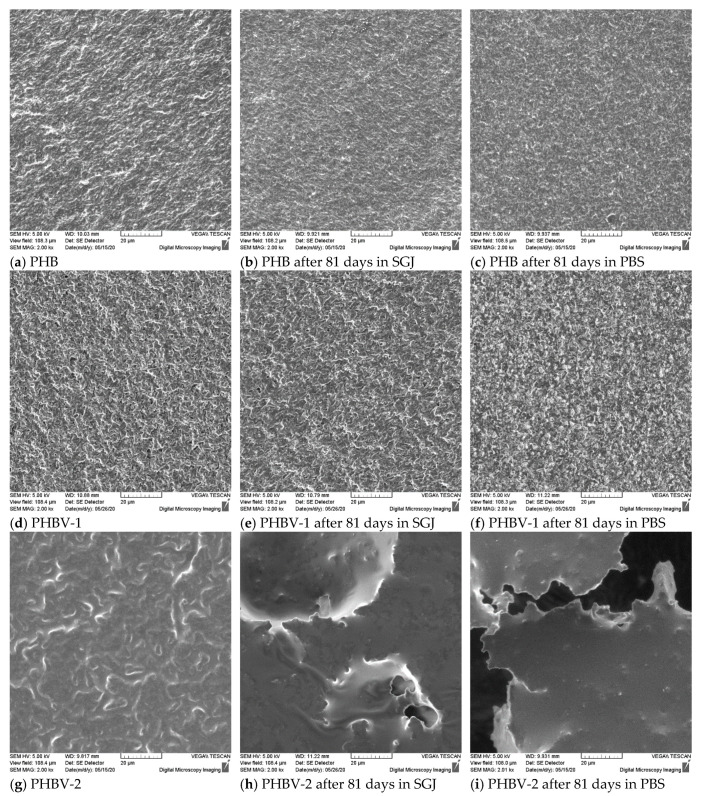
Morphological changes of original PHB, PHBV-1, and PHBV-2 and after 81 days degradation at 37 °C in synthetic gastric juice (SGJ) and phosphate buffer saline solution (PBS) with lipase.

The deterioration of scaffolds began on the surface and was closely related to the morphology of the samples. Figure 6 shows the morphology of the PHB, PHBV-1, and PHBV-2 scaffolds before and after 81 days of degradation in model fluids. The surface morphologies of the PHB (Figure 6a) and PHBV-1 (Figure 6d) scaffolds before degradation were very similar and could be described as a slightly grainy surface that is typical for highly crystalline polymers. In contrast, the surface of the PHBV-2 (Figure 6g) scaffolds was more plastic than the surfaces of the PHB and PHBV-1 scaffolds. This implies a significantly lower degree of crystallinity for the PHBV-2 sample that can be ascribed to the fact that PHBV-2 was not a pure copolymer but contained residual phenolics and polysaccharides of the grape sugar extract co-extracted from the biomass along with the PHA polymer. The effect of crystallinity on the visual appearance of the samples was much pronounced after degradation in model fluids. The morphology of the PHB and PHBV-1 scaffolds after 81 days of exposure in model fluids changed moderately; the surface morphology became only more structured. In contrast, the PHBV-2 scaffold’s morphology changed significantly, showing huge pits and craters (SGJ, Figure 6h) and torn structure (PBS, Figure 6i). Additionally, the surface became even smoother, which is typical for amorphous polymers (Figure 6h,i). Long-term exposure resulted in the “etching” of the outer thin layer, which was partially crystalline due to the boundary confinement leaving smooth amorphous phase exposed.

All tested samples displayed in Table 4 presented the thermal properties of scaffolds detected before and after degradation in model fluids. All samples before degradation displayed complex melting behavior with a first asymmetric small peak at 156.8−160.8 °C (T_m1_) and a secondary asymmetric peak at 172.7−173.8 °C (T_m2_). The double melting behavior in PHA may have indicated different physical characteristics of crystals, polymorphism, or the occurrence of a melting–recrystallization–remelting event [40]. The double melting behavior of the PHA scaffolds was also recorded after degradation in model fluids. After degradation in model fluids, the semi-crystalline character of the PHA scaffold was slightly modified. Some reorganization of the semi-crystalline nature could be seen from the slight changes in the values of melting temperatures and melting enthalpies.

The degree of crystallinity played an essential role in the PHA hydrolysis course. PHB and PHBV-1 had comparable values of melting enthalpies of 76.3 and 81.7 J g^−1^, respectively. PHBV-2 possessed a much lower melting enthalpy of 45.6 J g^−1^. The high degree of crystallinity of the samples caused a high inertness of the surfaces of the PHB and PHBV-1 scaffolds. In synthetic gastric juice, only PHBV-2 displayed a small decrease in the degree of crystallinity (the value of melting enthalpy decreased by about 11.4%). The values of melting enthalpies of PHB and PHBV-1 slightly increased, thus resulting in the recrystallization of amorphous parts. The degradation of the PHA scaffolds in PBS with lipase was more progressive and effective than synthetic gastric juice. Both scaffolds prepared from PHBV showed lower values of melting enthalpies after enzymatic degradation compared to values before degradation. PHBV-2 showed the most considerable decrease in melting enthalpy (about 29.6%) due to its much lower degree of crystallinity compared to PHB and PHBV-1. The results indicated that the extent of PHA surface degradation in the model fluids corresponded with the primary semi-crystalline character of the samples and the presence of an active enzyme.

The main principle of PHA degradation in fluids with or without enzymes is hydrolysis. The carboxylic esters start to decompose, resulting in a decrease in the polymer’s measured molecular weight. The variations in the molecular weight of PHAs during degradation were monitored using the SEC-MALLS technique (Table 5 and Table 6). SEC-MALLS chromatograms are shown in the Supporting Information (see Figures S1–S3). The values of weight-average molecular weight decreased the most in the case of PHB hydrolysis, showing around 51.7% and 43.7% losses in SGJ and PBS, respectively. The values of the average molecular weights of PHBV-1 and PHBV-2 after degradation were comparable, reaching a relative decrease in the range of about 33.6–37.1%.

**Figure 7:**
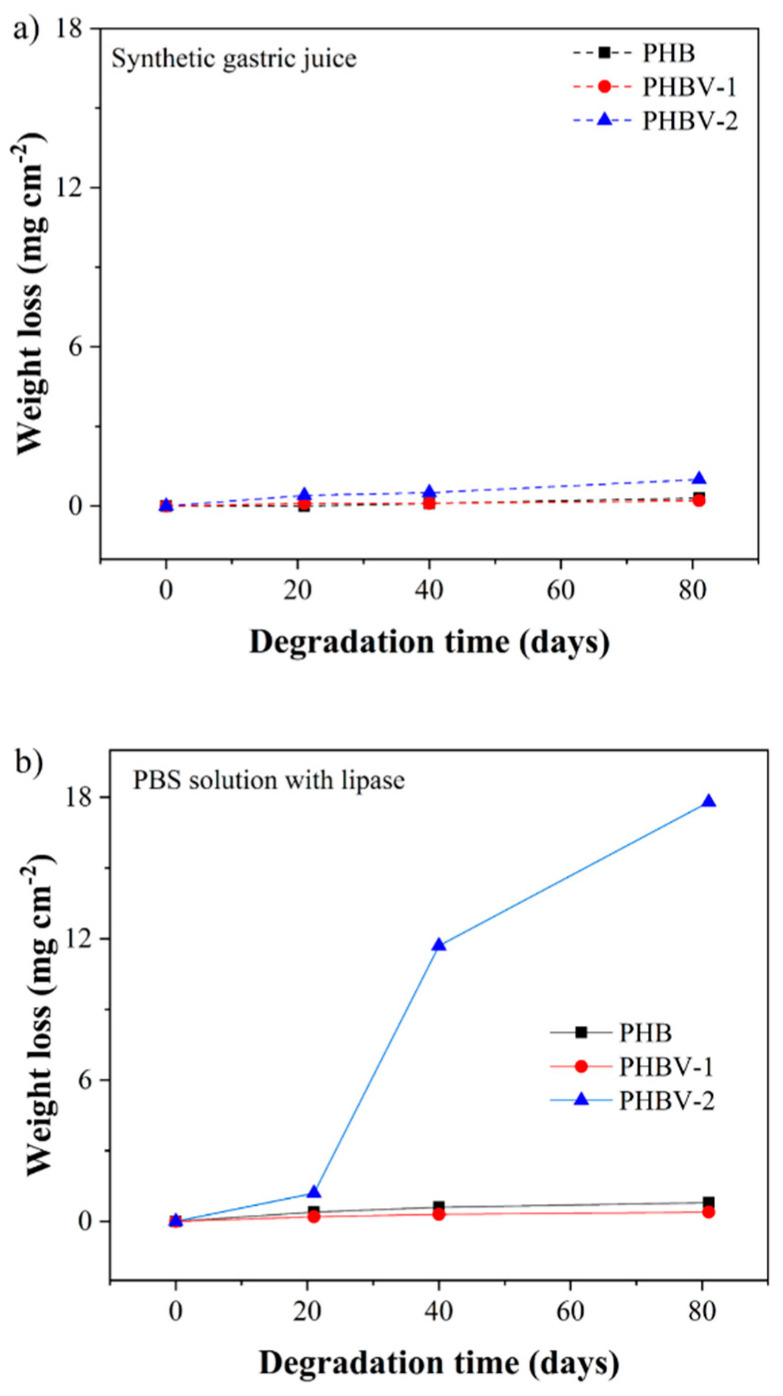
Degradation of PHB, PHBV-1, and PHBV-2 at 37 °C in (**a**) synthetic gastric juice and (**b**) phosphate buffer saline solution with lipase.

The changes that occurred in the scaffolds after 81 days in model fluids were assessed gravimetrically (Figure 7). The weight changes in all samples were only minor except for the PHBV-2 scaffold degraded in PBS buffer with lipase. The weight loss of PHBV-2 after 81 days in PBS buffer with lipase reached up to 85%.

## 4. Discussion

PHBV is a random copolymer of 3-hydroxybutyrate and 3-hydroxyvalerate. The copolymer with the increased 3HV concentration exhibited a higher flexibility, a lower melting point, and a lower crystallinity [41]. The determined percentage of 3HV presented in PHBV-1 and PHBV-2 was too small to influence their melting point compared to PHB. Moreover, since the 3HV fractions in PHBV-1 and PHBV-2 were similar, one would expect comparable thermal properties of both copolymers produced by employing the same bacterium using only various carbon sources. Therefore, it was surprising that the melting enthalpy of PHBV-2 was about 40.2% and 44.2% lower than in PHB and PHBV-1, respectively. The most likely reason for this was that PHBV-2 was not a pure copolymer but contains a small but essential portion of incorporated phenolics and polysaccharides, such as pectins and possibly other residues of grape sugar extract that were co-extracted from the biomass along with the PHA polymer. These substances did not affect the melting point of PHBV-2 but hindered its crystallization. The determined reduced crystallinity of PHBV-2 due to the incorporation of phenolics and pectin was consistent with the literature. Chan et al. prepared grafted pectin-PHB and found out that the polymer was fully amorphous. Additionally, the nanofibers prepared from pectin-PHB displayed a 39–335% enhancement of elongation at break compared to neat PHB fibers [42]. Consistent with this literature, the PHBV-2 scaffold was more flexible than the scaffolds made from PHB or PHBV-1 (Table 3).

**Figure 8:**
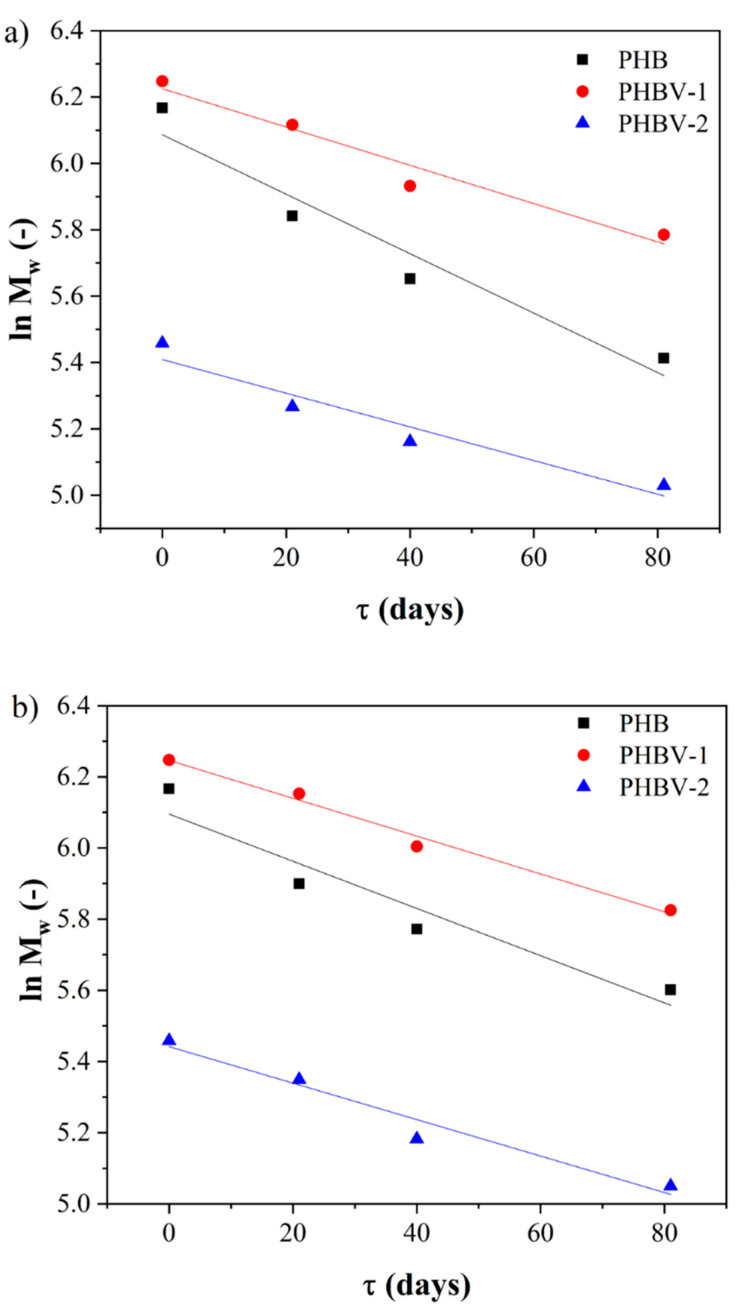
A linearized model of first-order kinetics applied on the degradation of PHB, PHBV-1, and PHBV-2 in (**a**) synthetic gastric solution and (**b**) phosphate buffer saline solution with lipase.

The hydrolysis and enzymatic degradation of PHAs are dependent on their chemical structure and physical properties such as morphology, sample dimensions, molecular weight, and crystallinity [43,44,45]. The degradation process of the PHA scaffolds was monitored by scanning electron microscopy, differential scanning calorimetry, SEC-MALLS, and gravimetrically. The most sensitive method indicating the degradation changes in the polymer occurring due to bond cleavage is considered to be the SEC-MALLS technique. Among the other essential parameters of polymer chemistry, the molecular weight represents the crucial feature reflecting PHA’s actual degradation state. It can also access the kinetics of degradation. This parameter’s importance is emphasized by its crucial role in other essential properties of PHAs, including mechanical properties, crystallinity, weight loss, and morphology [25]. The SEC-MALLS technique was used for the indication of the degradation state, the variation in polymer molecular weight, and the polydispersity of the studied scaffolds due to the bond cleavage of polymer chains. The comparison of the changes in values of molecular weight between the PHA scaffolds showed markedly higher losses in the PHB scaffolds than in the PHBV-1 or PHBV-2 scaffolds. The introduction of 3HV into the macromolecule enhanced the hydrolytic stability of the copolymer. The enhanced hydrolytic stability of the PHBV copolymers compared to PHB copolymers corresponded with the literature and may be explained by the higher hydrophobicity of PHBV than PHB [46]. The decrease of molecular weight in time for all PHA scaffolds showed typical exponential decay. Therefore, first-order kinetics for polymer degradation modeling could be applied (Figure 8).

The application of first-order kinetic model on the observed exponential decrease of molecular weight in time provided the value of k_D_ = (0.00896 ± 0.00160) day^−1^ for PHB, k_D_ = (0.00577 ± 0.00086) day^−1^ for PHBV-1, and k_D_ = (0.00507 ± 0.00097) day^−1^ for PHBV-2 (Table 7) for degradation in synthetic gastric juice. These values indicated the approximately three times lower degradation kinetics of the studied PHBV scaffolds than previously published poly(3-hydroxybutyrate-co-3-hydroxyhexanoate) copolymer degradation in the same environment [22]. The results summarized in Table 7 also indicate a comparable rate of degradation of the PHB and PHBV scaffolds in PBS with lipase.

These results indicated that the PHBV-2 scaffolds should have been the most stable material within the tested scaffolds. However, the detected weight changes after 81 days of hydrolysis in SGJ and PBS with lipase showed a different tendency. PHBV-2 was proved to be the most unstable material that lost about 14.5% and 95% weight in SGJ and PBS with lipase, respectively, after 81 days. Nevertheless, the weight losses determined for the PBV and PHBV-1 scaffolds in model fluids were only from 1.3% to 3.6%. On the other hand, the calculated values of k_D_ for the PHB and PHBV scaffold degradation must be considered with respect to the original starting molecular weight of the individual polymers. In this respect, PHBV-2 showed, from the beginning, a significantly lower molecular weight. The differences in the starting molecular weights of PHBV-2 to PHB and PHBV-1 were 51.1% and 54.5%, respectively, which were considerably higher than the difference in the calculated k_D_ for PHB and PHBV copolymers.

Similarly, SEM analysis confirmed visible morphological changes the PHBV-2 scaffolds’ surfaces (Figure 6). The reason for such material destruction was the high proportion of the amorphous part in the PHBV-2 material. It is well-documented that amorphous materials are substantially more prone to enzymatic and abiotic degradation [47]. Therefore, surface erosion took significant part in the degradation. The observed degradation tendency of the PHBV-2 scaffolds in PBS with lipase compared to the PHB and PHBV-1 scaffolds corresponded with other studies showing that first are hydrolyzed PHB chains in an amorphous state.

Moreover, the degradation process is accelerated by the presence of suitable hydrolytic enzymes [48,49]. The crystalline/amorphous portion is an important parameter that affects the predisposition of polyesters to undergo enzyme hydrolysis. Wu et al. observed that an amorphous copolymer of 3-hydroxybutyrate and 4-hydroxybutyrate is much more susceptible to hydrolysis by lipase than a highly crystalline homopolymer of 3-hydroxybutyrate [9]. Finally, two main degradation ways of polyesters in fluids can be followed. The first one is the bond cleavage known as a bulk erosion, and the second one is a depletion of material that occurs due to surface erosion. The bulk erosion results in the loss of molecular weight, and surface erosion supports material weight loss [50]. Our results clearly illustrated that even a small portion of “impurities,” associated with PHA production and substrate used, might dramatically affect the properties of produced materials and their applicability. Even though PHBV-1 and PHBV-2 contained very similar 3HV fractions, their properties and their usability differed substantially. PHBV-1 is more suitable for scaffolds requiring more rigid and resistant materials; on the other hand, the PHBV-2 material seems usable if the more flexible and biodegradable nature of the material is welcome. Of course, the effect of “impurities” present in PHBV-2 should be analyzed in detail in our future work to test their potential toxicity or other harmful properties, which might be a disadvantage in tissue engineering. However, theoretically, phenolics and pectins are considered as “green” and safe molecules, which may modify crystallinity of PHAs.

## 5. Conclusions

The present study reports the biosynthesis of PHBV copolymers in 2-L bioreactors by using fructose or grape sugar extracts as carbon sources for the cultivation of *Cupriavidus necator*. Grape sugar extract was extracted from waste grape pomace and, as the carbon source for PHA producers, was transformed into high-value products—PHBV. The production yields of PHBV-2 using the grape sugar extract were lower than the PHBV-1 yields in the case of using fructose. Though both polymers contained very similar 3HV fractions, their properties differed substantially, which might be attributed to the presence of phenolics and pectins, co-isolated with PHA materials produced on grape sugar extract. Generally, incorporation of these “impurities” substantially reduced the crystallinity and improved the flexibility of the material.

The study of hydrolysis in synthetic gastric juice and enzymatic hydrolysis in PBS with lipase showed that the degradation of the PHBV-2 scaffolds had different character compared to those PHBV-1 and PHB scaffolds. The bulk degradation of both the PHBV-1 and PHBV-2 scaffolds was much slower compared to the PHB scaffolds. However, the main differences occurred due to surface erosion. The PHBV-2 scaffolds had significantly lower crystallinity and brittleness compared to the PHBV-1 and PHB scaffolds. The surface erosion of PHBV-2 was much faster than in the case of PHBV-1. The most apparent degradation of the PHBV-2 scaffolds was recorded in PBS with lipase after 81 days.

In conclusion, the present study has highlighted the utilization of lignocellulosic waste by-products and grape pomace as carbon sources for PHA biosynthesis. The results illustrated that the use of waste grape pomace as a carbon source for PHA production might contribute not only to the economy of the biosynthesis of biopolymers but also the modification of PHBV properties.

## Figures and Tables

**Table 1:** Cultivation of *C. necator* and poly(3-hydroxybutyrate-co-3-hydroxyvalerate) (PHBV) after 36 h production on fructose and lyophilized grape sugar extract. PHBV-1: *Cupriavidus necator* grown on fructose; PHBV-2: *Cupriavidus necator* grown on grape sugar; CDW: cell dry weight; PHA: polyhydroxyalkanoate; 3HV: 3-hydroxyvalerate fraction.

Sample	Substrate	CDW (g L^−1^)	PHA (g L^−1^)	PHA (%)	3HV Fraction (%)
PHBV-1	fructose	14.6	5.3	36.3	13.3
PHBV-2	grape sugar	20.8	3.5	16.8	9.7

**Table 2:** Attributions of the main infrared peaks of the poly(3-hydroxybutyrate) (PHB) and PHBV copolymers.

Wavenumber (cm^−1^)	Correspondence
2970	asymmetric C–H stretching of methyl groups
2938	symmetric C–H stretching of methyl groups
1722	symmetric C=O stretching of aliphatic ester (crystalline phase of PHB and PHBV copolymers)
1453	asymmetric C–H bending of methyl and methylene groups
1379	symmetric C–H bending of methyl groups (umbrella mode vibration of –CH_3_ groups)
1276	symmetric C–O stretching of aliphatic ester (crystalline phase of PHB and PHBV copolymers)
1261	symmetric C–O stretching of aliphatic ester
1225	symmetric C–O stretching of aliphatic ester (crystalline phase of PHB and PHBV copolymers)
1178	asymmetric C–O–C stretching of aliphatic ester (amorphous phase of PHB and PHBV copolymers)
1130	symmetric C–O–C stretching of aliphatic ester (amorphous phase of PHB and PHBV copolymers)
1100	asymmetric O–C–C stretching of saturated aliphatic ester
1053	O–C–C stretching of saturated aliphatic ester (stretching of the second C–O bond in the aliphatic ester, which is the one to the right of the ester oxygen)
980	skeletal C–C vibration mode (crystalline phase of PHB and PHBV copolymers)

**Table 3:** Crystallinity index and mechanical behavior of the PHB, PHBV-1, and PHBV-2 scaffolds. (average value ± SD, n = 5). *I*_C–O_/*I*_–CH2–_: the ratio of the intensity at 1225 cm^−1^ to the intensity at 1453 cm^−1^.

Sample	*I*_C–O_/*I*_–CH2–_ (–)	E-Modulus (MPa)	σ_B_ (MPa)	ε_B_ (%)
PHB	3.87	1830 ± 42	33 ± 2.0	8.4 ± 1.7
PHBV-1	1.42	1124 ± 38	23.1 ± 1.2	25.1 ± 1.4
PHBV-2	1.12	649 ± 54	10.2 ± 3.2	48.0 ± 8.2

**Table 4:** Thermal characteristics derived from the first DSC heating scan.

Sample	Before Degradation			After 81 Days in Synthetic Gastric Juice			After 81 Days in PBS with Lipase		
	T_m1_ (°C)	T_m2_ (°C)	ΔH_m_ (J g^−1^)	T_m1_ (°C)	T_m2_ (°C)	ΔH_m_ (J g^−1^)	T_m1_ (°C)	T_m2_ (°C)	ΔH_m_ (J g^−1^)
PHB	158.5	173.6	76.3	158.8	172.4	83.1	159.1	174.0	79.4
PHBV-1	160.8	173.8	81.7	161.1	173.8	89.3	163.0	175.7	78.8
PHBV-2	156.8	172.7	45.6	155.9	171.8	40.4	155.9	172.7	32.1

**Table 5:** Molecular weight changes in the PHA scaffolds in vitro at 37 °C in synthetic gastric juice determined by SEC-MALLS. Đ: polydispersity.

Incubation Time (Days)	M_w_ (kDa)	Đ	Relative Decrease of M_w_ (%)
PHB	−	−	−
0	480 ± 4.22	1.50 ± 0.05	0
21	344 ± 2.07	1.32 ± 0.01	28.3
40	285 ± 4.74	1.31 ± 0.06	40.6
81	232 ± 7.06	1.24 ± 0.01	51.7
PHBV_1	−	−	−
0	517 ± 11.08	1.06 ± 0.02	0
21	453 ± 1.08	1.18 ± 0.04	12.4
40	371 ± 6.21	1.27 ± 0.05	28.2
81	325 ± 8.13	1.17 ± 0.04	37.1
PHBV_2	−	−	−
0	235 ± 5.33	1.26 ± 0.05	0
21	194 ± 4.25	1.16 ± 0.04	17.4
40	170 ± 6.82	1.11 ± 0.01	27.6
81	155 ± 2.34	1.05 ± 0.01	34.0

**Table 6:** Molecular weight changes in the PHA scaffolds in vitro at 37 °C in a phosphate buffer saline solution with lipase determined by SEC-MALLS.

Incubation Time (Days)	M_w_ (kDa)	Đ	Relative Decrease of M_w_ (%)
PHB	−	−	−
0	480 ± 4.22	1.50 ± 0.05	0
21	365 ± 4.86	1.29 ± 0.02	23.9
40	323 ± 1.73	1.19 ± 0.01	32.7
81	270 ± 2.19	1.19 ± 0.04	43.7
PHBV_1	−	−	−
0	517 ± 11.08	1.06 ± 0.02	0
21	470 ± 3.00	1.17 ± 0.04	9.1
40	399 ± 5.11	1.20 ± 0.05	22.8
81	339 ± 5.17	1.22 ± 0.09	34.4
PHBV_2	−	−	−
0	235 ± 5.33	1.26 ± 0.05	0
21	210 ± 0.81	1.18 ± 0.01	10.6
40	179 ± 1.56	1.14 ± 0.02	23.8
81	156 ± 2.54	1.07 ± 0.01	33.6

**Table 7:** Kinetic model parameters and coefficients of determinations (R2) for the degradation of PHB, PHBV-1, and PHBV-2 in model fluids at 37 °C.

Sample	Synthetic Gastric Solution		PBS with Lipase	
	k_D_ (day^−1^)	R^2^	k_D_ (day^−1)^	R^2^
PHB	0.00896 ± 0.00160	0.91051	0.00663 ± 0.00138	0.88034
PHBV-1	0.00577 ± 0.00086	0.93621	0.00532 ± 0.00042	0.98132
PHBV-2	0.00507 ± 0.00097	0.89771	0.00512 ± 0.00075	0.93831

## Supplementary Materials

The following are available online at https://www.mdpi.com/1996-1944/13/13/2992/s1, Figure S1: SEC-MALLS chromatograms obtained for PHB degradation in (A) PBS with lipase, (B) SGJ. Figure S2: SEC-MALLS chromatograms obtained for PHBV-1 degradation in (A) PBS with lipase, (B) SGJ. Figure S3: SEC-MALLS chromatograms obtained for PHBV-2 degradation in (A) PBS with lipase, (B) SGJ.
