# Supplementary material for: Enzymatic Hydrolysis of Poly(3-Hydroxybutyrate-co-3-Hydroxyvalerate) Scaffolds
Source: Materials (Basel). 2020 Jul 5;13(13):2992. doi: 10.3390/ma13132992 (PMC7372466; doi:10.3390/ma13132992)
Supplement: Supplementary file 1 [file materials-13-02992-s001.pdf]

materials

Supplementary Materials

Enzymatic Hydrolysis of Poly(3-Hydroxybutyrate-*co*-3-Hydroxyvalerate) Scaffolds

Adriana Kovalcik [1,*], Stanislav Obruca [1], Michal Kalina [2], Michal Machovsky [3], Vojtech Enev [2], Michaela Jakesova [1], Marketa Sobkova [1] and Ivana Marova [1]

[1] Department of Food Chemistry and Biotechnology, Faculty of Chemistry, Brno University of Technology, Purkynova 118, 612 00 Brno, Czech Republic; obruca@fch.vut.cz (S.O.); xcjakesova@vutbr.cz (M.J.); xcsobkovama@vutbr.cz (M.S.); marova@fch.vut.cz (I.M.)

[2] Department of Physical and Applied Chemistry, Faculty of Chemistry, Brno University of Technology, Purkynova 118, 612 00 Brno, Czech Republic; kalina-m@fch.vut.cz (M.K.); enev@fch.vut.cz (V.E.)

[3] Centre of Polymer Systems, Tomas Bata University in Zlin, trida Tomase Bati 5678, 760 01 Zlin, Czech Republic; machovsky@utb.cz

* Correspondence: kovalcik@fch.vut.cz; Tel.: +420-541-149-422

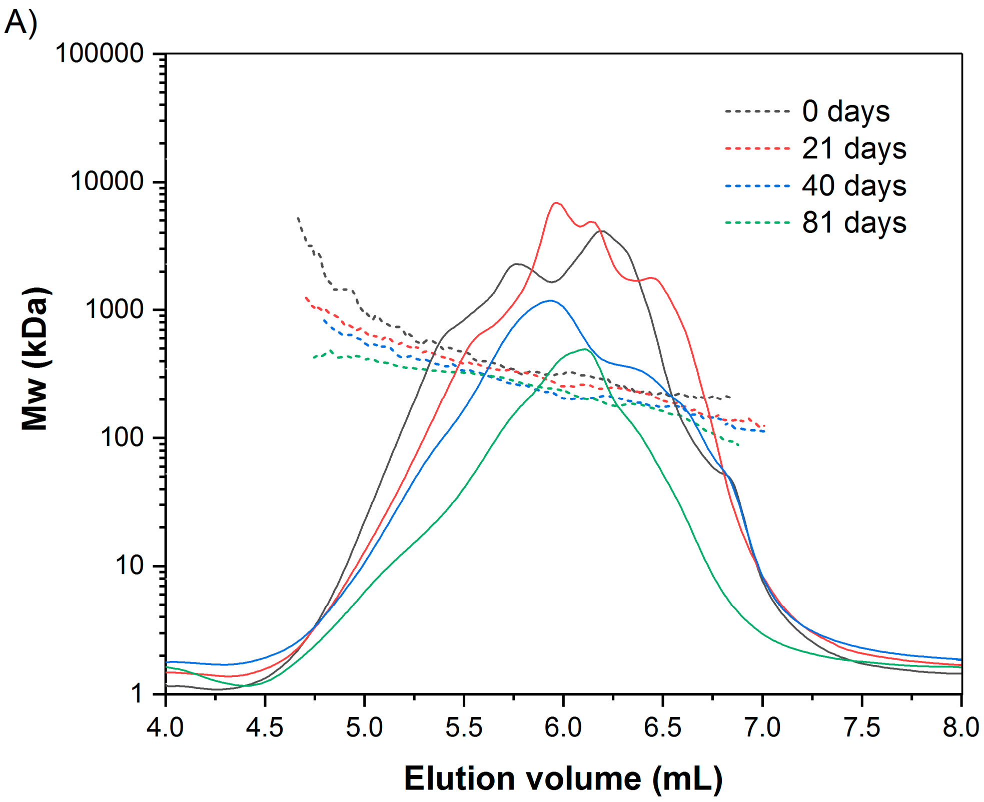

B)

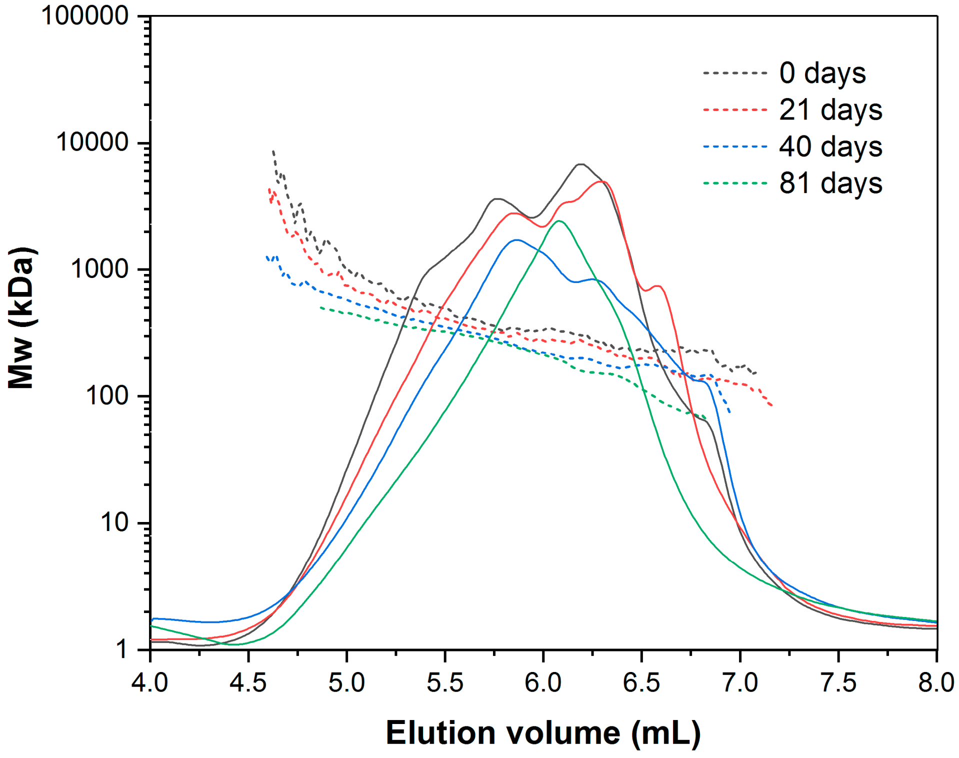

Figure S1. SEC-MALLS chromatograms obtained for PHB degradation in (**A**) PBS with lipase, (**B**) SGJ.

A)

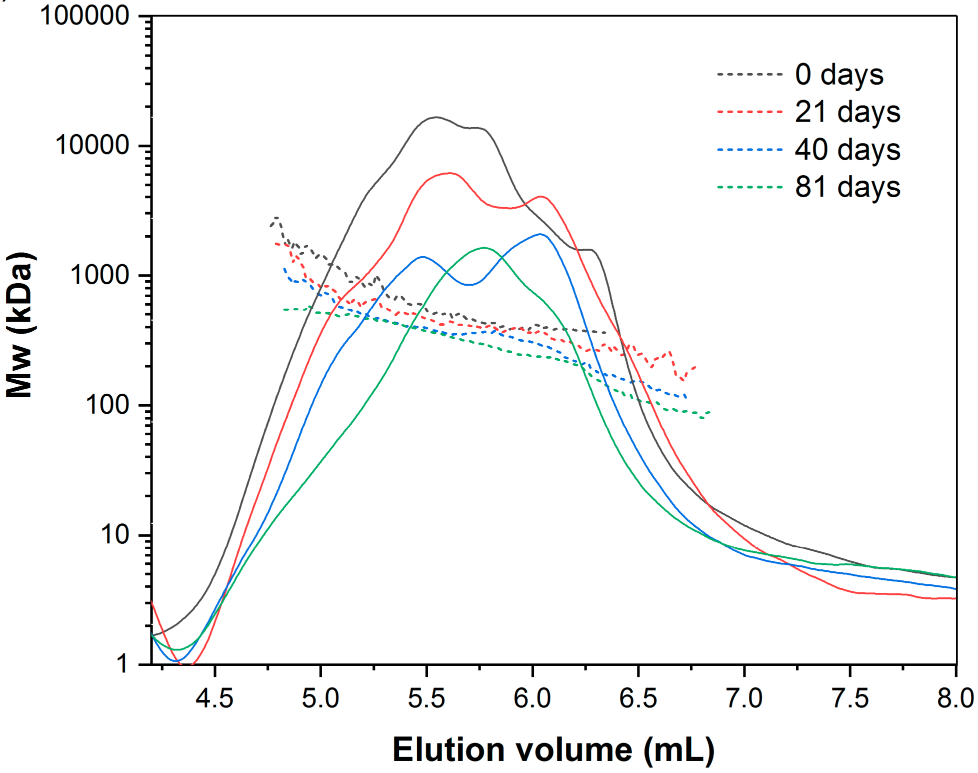

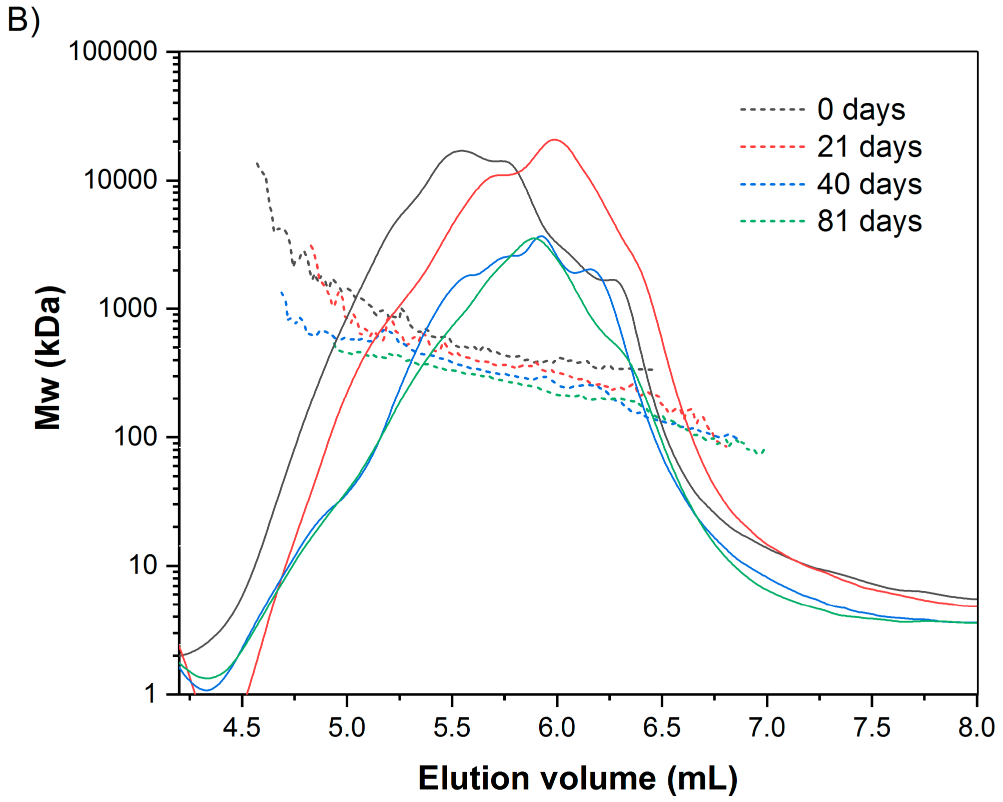

Figure S2. SEC-MALLS chromatograms obtained for PHBV-1 degradation in (**A**) PBS with lipase, (**B**) SGJ.

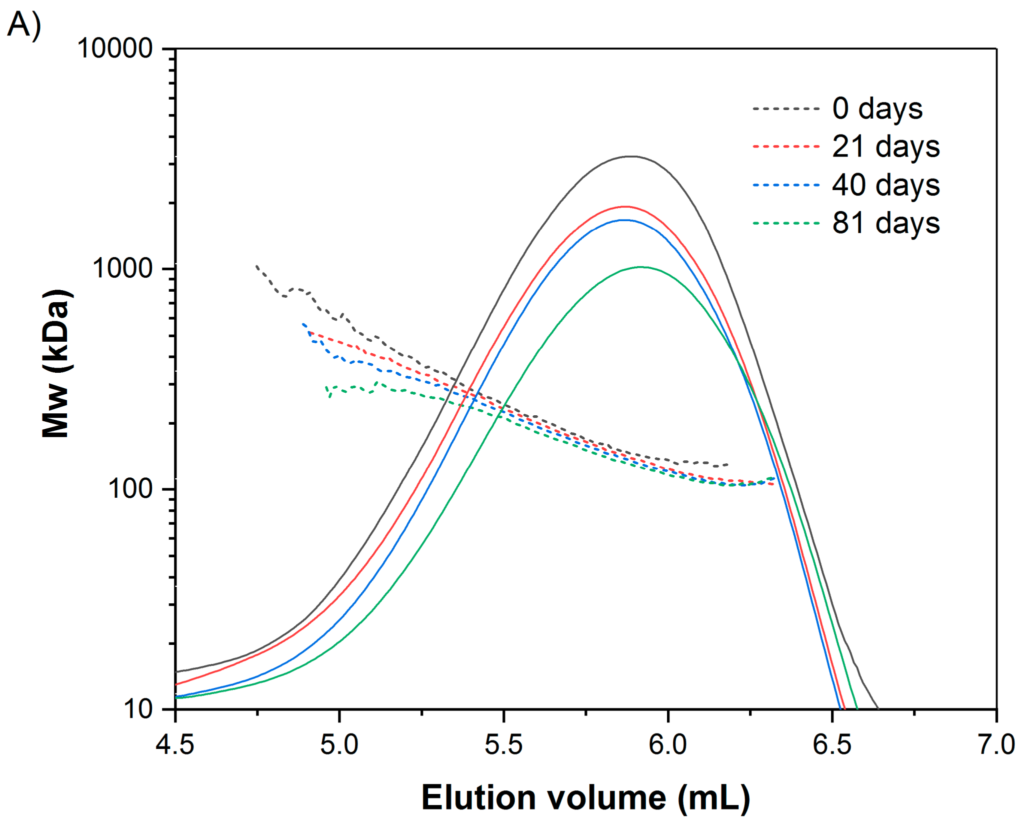

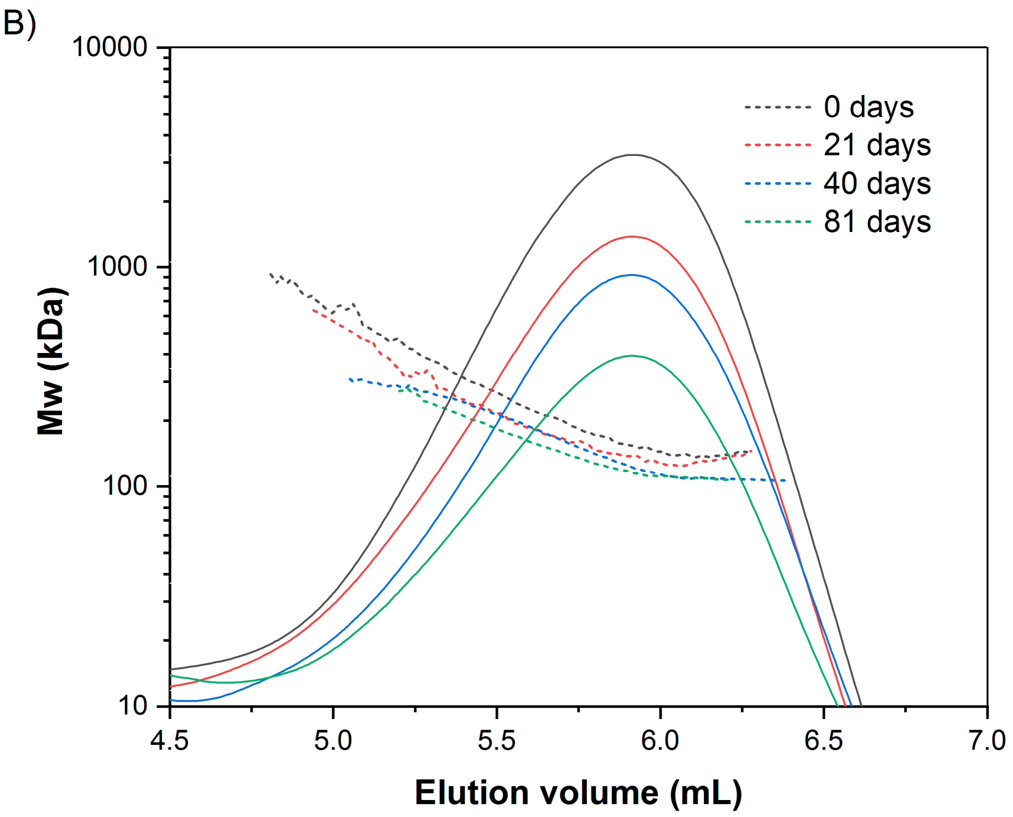

Figure S3. SEC-MALLS chromatograms obtained for PHBV-2 degradation in (**A**) PBS with lipase, (**B**) SGJ.

© 2020 by the authors. Submitted for possible open access publication under the terms and conditions of the Creative Commons Attribution (CC BY) license (http://creativecommons.org/licenses/by/4.0/).
